# Improving the Mechanical and Electrical Properties of Ceramizable Silicone Rubber/Halloysite Composites and Their Ceramic Residues by Incorporation of Different Borates

**DOI:** 10.3390/polym10040388

**Published:** 2018-04-01

**Authors:** Jianhua Guo, Xuming Chen, Yong Zhang

**Affiliations:** 1 School of Materials Science and Engineering, South China University of Technology, Guangzhou 510640, China; chenxm233@126.com (X.C.); tropo@foxmail.com (Y.Z.); 2 Institute of Modern Industrial Technology of SCUT in Zhongshan, Zhongshan 528400, China

**Keywords:** ceramizable silicone rubber, borate, halloysite, composite, ceramizable mechanism

## Abstract

Ceramizable silicone rubber (MVQ)/halloysite (HNT) composites were fabricated by incorporation of three different borates, including sodium tetraborate decahydrate, ammonium pentaborate, and zinc borate into MVQ matrix, respectively. The composites without any borates were also prepared as control. The effect of the borates on the mechanical and electrical properties of MVQ/HNT composites was investigated. The ceramic residues were obtained from the decomposition of the composites after sintering at 1000 °C. The effect of the borates on the linear shrinkage, weight loss, and flexural and impact strength of the residues was also studied. The fracture surfaces of the composites and their corresponding residues were observed by SEM. The proposed ceramizable mechanism of the composites by incorporation of different borates was revealed by XRD analysis.

## 1. Introduction

Ceramizable silicone rubber (MVQ)-based composites have good processability like common elastomers at room temperature and transform into hard ceramic residues in fire environments or at elevated temperatures, which are desired materials to fulfill the increasing requirements in special applications such as fire resistant wires and cables [1]. Commonly, ceramizable silicone rubber-based composites are composed by polysiloxane elastomer, mineral fillers (montmorillonite, kaolin, talc, mica, etc.) [2,3,4,5,6,7], fluxing agents (glass frit, ammonium polyphosphate, etc.) [8,9,10], and other additives. Herein, fluxing agents are important components for ceramizable MVQ-based composites, which will be melted at elevated temperatures to merge some residues derived from the pyrolysis products of the composites. Furthermore, fluxing agent can promote low temperature ceramization and improve the mechanical strength of the ceramic residues [11,12,13]. At present, many studies have been conducted on the properties of ceramizable MVQ-based composites with the addition of glass frits with low softening point temperature. For example, Mansouri et al. [14] found that silicone rubber by incorporation of 2.5 wt % glass frits with a softening point temperature of 525 °C can increase the flexural strength of the residues from 0.42 to 2.54 MPa after firing at 800 °C. Guo et al. [15] showed that the flexural strength of ceramic residues after sintering at 1000 °C increased from 2 to 13.3 MPa when the loading of glass frits with a softening point temperature of 480 °C increased from 0 to 30 phr. However, the addition of glass frits will lead to a decrease of thermal stability of silicone rubber due to the catalyzed degradation effect of metal oxides decomposed from the glass frits [16]. Thus, some other fluxing agents except for glass frits are urgently needed to prepare ceramizable MVQ-based composites.

It was reported that zinc borate (ZB) can act as flame retardant, smoke suppressant, and antitracking agent in both halogen-containing and halogen-free polymers [17]. The presence of ZB can also result in a significant char residue formation. Moreover, ZB plays a role of glass phase, helping to form a bridge between polymer matrix and mineral fillers, which is used extensively in polyvinyl chloride, polyamide, polyolefin, epoxy, phenolic, and poly(vinyl acetate) (PVAc) [18]. As we know that boron oxide (B_2_O_3_) will be generated by the decomposition of zinc borate after heating, which provides afterglow suppression in polymers. Moreover, B_2_O_3_ will form glassy protective surface at 325 °C, which can act as a fluxing agent [19]. For example, Anyszka et al. [20] showed that the compressive strength of ceramic residues after firing at 1000 °C increased by more than four times when the loading of B_2_O_3_ increased from 20 to 40 phr. Halloysite nanotubes (HNTs) is a natural silicate mineral, consisting of 1:1 aluminosilicate layers with a tubular structure. Due to their special morphology, chemical structure, and surface properties, HNTs are widely used in fine ceramics [21], supported nanoparticles [22], and polymer composites [23]. However, HNTs acting as ceramic fillers and different borates acting as fluxing agents applied in MVQ have not been investigated in detail.

In this study, MVQ/HNT composites by incorporation of sodium tetraborate decahydrate, ammonium pentaborate, and zinc borate respectively, were fabricated and the composites without any fluxing agents were also prepared as control. The effect of the borates on the mechanical and electrical properties of ceramizable MVQ/HNT composites and the flexural and impact strength of the ceramic residues were investigated. Furthermore, the morphology of the ceramic residues was characterized by digital camera and SEM, and the proposed ceramic mechanism of MVQ-based composites was revealed by XRD.

## 2. Experimental

### 2.1. Materials

Methyl vinyl silicone rubber (MVQ, model 110-2, with a molecular weight of 600,000) was purchased from Caiyan Co., Ltd., Guangdong, China. Fumed silica (CABOSIL M-5) was purchased from Cabot Co., Ltd., Boston, MA, USA. Halloysites (HNTs) have a length in the range of 0.5–3.0 microns, an exterior diameter in the range of 50–70 nanometers and an internal diameter (lumen) in the range of 15–30 nanometers. HNTs were produced by Linshounanyi Mineral Factory, Hebei, China. Sodium tetraborate decahydrate (Na_2_B_4_O_7_·10H_2_O) was purchased from Fuchen Chemical Reagent Factory, Tianjin, China. Ammonium pentaborate (NH_4_B_5_O_8_·4H_2_O) was purchased from Maclin Biochemical Sci. & Tech. Co., Ltd., Shanghai, China. Zinc borate (2ZnO·3B_2_O_3_·3.5H_2_O) was purchased from Guanghua Sci. & Tech. Co., Ltd., Guangdong, China. Calcium carbonate was supplied by Enping Yanhua Industrial Co., Ltd., Guangdong, China. 2,5-dimethyl-2,5-bis (tert butyl peroxy) hexane (DBPMH, model C-15) was supplied by Caiyuan Silicone Materials Co., Ltd., Guangdong, China. The formulations of MVQ/HNT composites are summarized in Table 1. The formulation symbols are labeled as NaB, NHB and ZB for the composites by incorporation of sodium tetraborate decahydrate, ammonium pentaborate, and zinc borate, respectively.

### 2.2. Preparation of Silicone Rubber Based Ceramizable Composites

All of the compounds were prepared on a two-roll mill (model XK-160, Zhanjiang Machinery Factory, Guangdong, China) with a gear ratio of 1:1.4 at room temperature. The polysiloxane elastomer was first softened, and then the fumed silica, calcium carbonate, halloysites, and borates were added until a visually good dispersion was achieved. The curing agent DBPMH was then added and processed until a homogenous batch was obtained. The total mixing time is about 20 min. The silicone rubber compounds were cured with flat sheets by compression molding in a hydraulic press (Model KSHR100T, Kesheng Industrial Co., Ltd., Guangdong, China) at 170 °C for 15 min under 15 MPa pressure. The post curing of samples was carried out in an oven at 180 °C for 2 h. The ceramizing of flat sheet samples (50 mm long, 4 mm thick, and 6mm wide) was performed using a muffle furnace (Shenjia KL-12B, Luoyang, China). The samples were heated from room temperature to 1000 °C at a heating rate of 10 °C·min^−1^, held for 30 min, and then cooled with the muffle furnace.

### 2.3. Characterization of the Composites and Ceramic Residues

The flexural strength of the ceramic residues was determined by a flexural method using a universal testing machine (Zwick/Roell Z010, Ulm, Germany) with a cross-head speed of 0.5 mm·min^−1^ according to ASTM D790-10. The impact strength was evaluated on an electronic Charpy impact tester (Suns, Shenzhen, China) according to ASTM D256-10e1. The weight loss of the ceramic residues was calculated according to Equation (1), where *W* is the weight loss, and *m*_1_ and *m*_2_ are the mass of the samples before and after sintering, respectively.

*W* = (*m*_1_ − *m*_2_)/*m*_1_ × 100% (1)

The shrinkage of the ceramic residue was measured by heating flat sheet samples to 1000 °C for 30 min in a muffle furnace. The linear shrinkage of the ceramic residue was calculated according to Equation (2), where *L* is the linear shrinkage, and *l*_1_ and *l*_2_ are the length of the samples before and after sintering, respectively.

*L* = (*l*_1_ − *l*_2_)/*l*_2_ × 100% (2)

The surfaces of the ceramizable MVQ/HNT composites and corresponding ceramic residues were observed by a digital camera (Canon 600D, Tokyo, Japan). The tensile fracture surface of the composites and the impact fracture surface of the ceramic residues were sprayed with gold and then observed by scanning electron microscopy (SEM, Zeiss EVO18, Oberkochen, Germany). Thermal gravity analysis (TGA) was performed by using TG 309F1 instrument (Netzsch, Selb, Germany). Samples (weight 8~10 mg) were heated at a heating rate of 10 °C·min^-1^ from 30 to 900 °C under nitrogen atmosphere. X-ray diffraction spectra of the ceramic residues were obtained using an X-ray diffractometer (XRD, PANlytical X’pert, Almelo, The Netherlands). Each scan was conducted from a *2*θ angle of 5°~75° at a scan rate of 1°·min^−1^.

## 3. Results and Discussions

### 3.1. Characterization of the Borates

**Figure 1:**
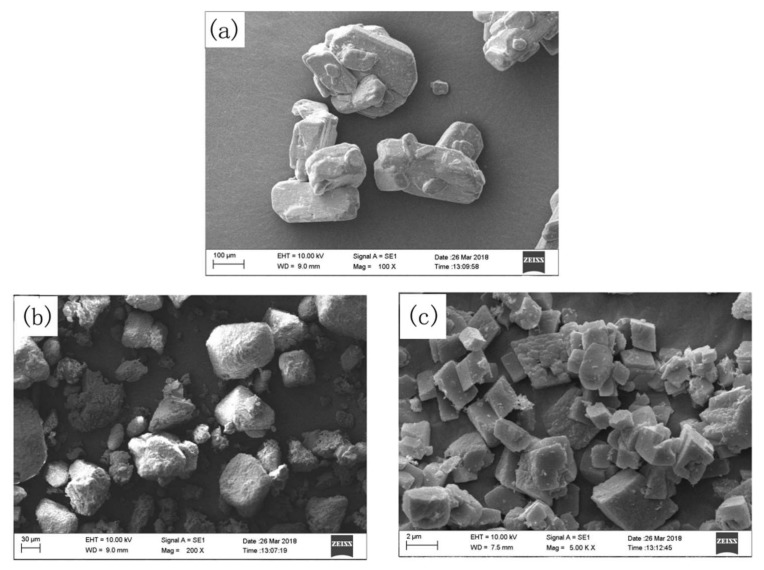
SEM photographs of (**a**) sodium tetraborate decahydrate, (**b**) ammonium pentaborate, and (**c**) zinc borate.

Halloysites (HNTs) act as the ceramic fillers in the MVQ composites, with the similar function as kaolin, mica, talc, etc. Some borates including sodium tetraborate decahydrate (NaB), ammonium pentaborate (NHB), and zinc borate (ZB), serve as the fluxing agents. The SEM photographs of three borates were showed in Figure 1. It can be observed in Figure 1a that the NaB particles are clustered at certain area and form aggregates. The average size of the NaB particles is about 200 μm. It is found that the NHB particles are spherical in shape with an average diameter of 50 μm (Figure 1b). However, the ZB particles have sharper edges than the NaB and NHB particles as depicted in Figure 1c. The ZB particles are irregular with a much smaller size of about 1~3 μm. 

**Figure 2:**
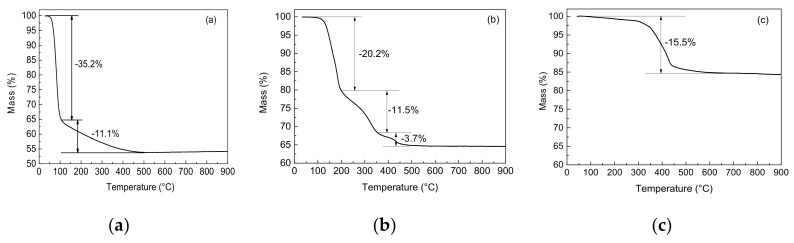
TGA curves of (**a**) sodium tetraborate decahydrate, (**b**) ammonium pentaborate, and (**c**) zinc borate.

Thermal stability of sodium tetraborate decahydrate, ammonium pentaborate, and zinc borate was investigated in Figure 2, and the corresponding thermal parameters are listed in Table 2. It is shown that the initial decomposition temperature (T_5%_) of sodium tetraborate decahydrate, ammonium pentaborate, and zinc borate are 66.9, 144.0, and 371.5 °C, respectively. The maximum degradation temperature (T_d%_) of zinc borate is much higher than that of sodium tetraborate decahydrate and ammonium pentaborate. The mass of the residues obtained from the decomposition of sodium tetraborate decahydrate, ammonium pentaborate and zinc borate at 900 °C is 54.2, 64.6, and 84.3%, respectively. Zinc borate, sodium tetraborate decahydrate, and ammonium pentaborate have one, two, and three decomposition process, respectively. The mass loss of first decomposition of sodium tetraborate decahydrate, ammonium pentaborate and zinc borate is 35.2, 20.2 and 15.5% respectively, which is attributed to the removal of crystal water within their chemical structure. Thus, zinc borate shows better thermal stability than sodium tetraborate decahydrate and ammonium pentaborate.

### 3.2. Effect of Borates on the Mechanical Properties of Ceramizable MVQ/HNT Composites

**Figure 3:**
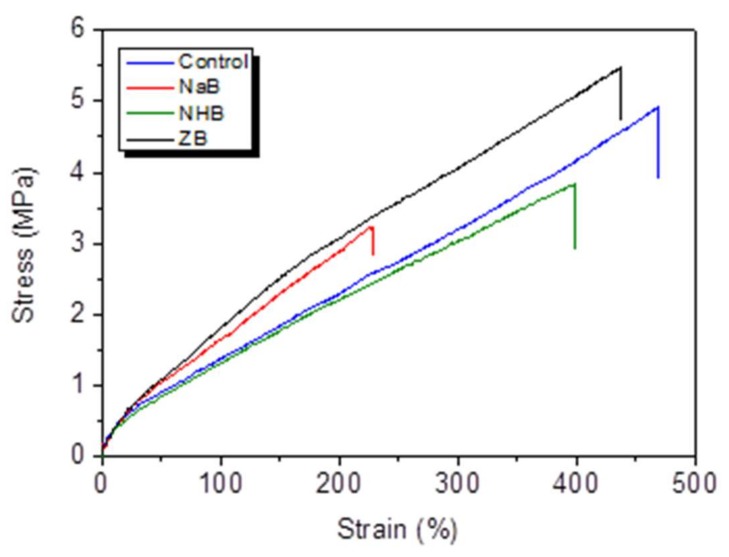
Tensile stress–strain curves of ceramizable MVQ/HNT composites with the addition of different borates.

The tensile stress–strain curves of ceramizable MVQ-based composites were shown in Figure 3. For the control, the tensile strength and elongation at break are 4.9 MPa and 469%, respectively. The tensile strength and elongation at break of the composites with the addition of sodium tetraborate decahydrate (abbr. as NaB composite) and ammonium pentaborate (abbr. as NHB composite) decrease, compared with the control. However, the tensile strength of the MVQ/HNT composite by incorporation of zinc borate (abbr. as ZB composite) is 5.5 MPa, which is 72% and 45% higher than that of the NaB and NHB composites respectively. According to the TGA results in Figure 2, the thermal decomposition of NaB and NHB fillers leads to the releasing of water steam during the curing process of the MVQ composites at 170 °C to form some voids or flaws in the MVQ matrix. The voids will result in a decrease in the mechanical properties of the MVQ composites. However, because of the higher initial decomposition temperature of ZB, it is easier to form dense structure in the MVQ composites than another two borates. Furthermore, another reason is that ZB in the composites might cause an increase in the physical crosslinking centers due to the higher electronegativity of divalent zinc ions than other elements in the blend [24], leading to an increase in the mechanical properties.

### 3.3. Effect of Borates on the Electrical Properties of Ceramizable MVQ/HNT Composites

**Figure 4:**
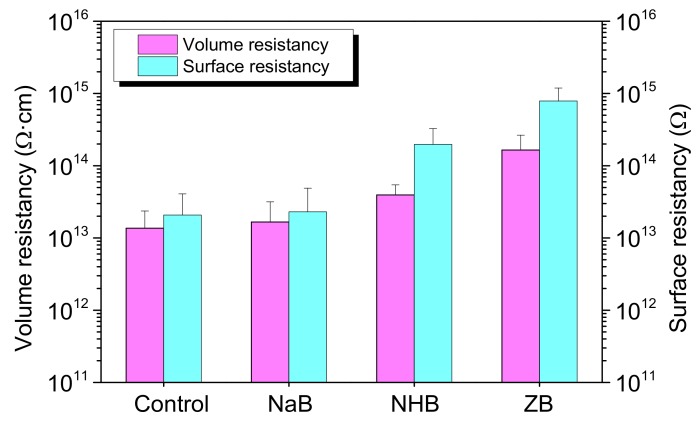
Electrical insulation properties of ceramizable MVQ/HNT composites with the addition of different borates.

The volume and surface resistivity of ceramizable MVQ/HNT composites with different borates were shown in Figure 4. For the control, the volume and surface resistivity are 1.36 × 10^13^ Ω·m and 2.08 × 10^13^ Ω, respectively. By incorporation of the borates, the electrical resistivity of the composites increase to some extent. Particularly, the ZB composite shows the maximum volume and surface resistivity with the values of 1.65 × 10^14^ Ω·m and 7.88 × 10^14^ Ω respectively, indicating that zinc borate will significantly improve the electrical insulation properties of MVQ. Similarly, zinc borate can improve the electrical insulation properties of ethylene–vinyl acetate copolymer (EVA)/metal hydroxide systems [17] and linear low-density polyethylene/rubbers/magnesium oxide composite [24]. These results may be attributed to collision of electrons by ZB particles inside the matrix which would obstruct the electron avalanche in the composites, and more obstruction would be generated finally resulting in higher volume and surface resistivity [24]. 

### 3.4. Effect of Borates on Linear Shrinkage and Weight Loss of Ceramic Residues

**Figure 5:**
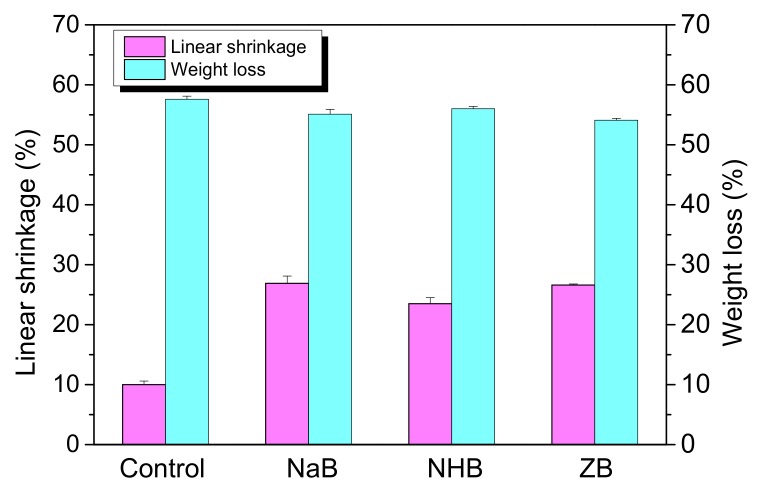
Linear shrinkage and weight loss of the ceramic residues by incorporation of different borates.

Linear shrinkage and weight loss of the ceramic residues obtained from the decomposition of the MVQ-based composites by incorporation of different borates were shown in Figure 5. The linear shrinkage of ceramic residues with the addition of different borates is bigger than that of the control because B_2_O_3_ can act as a fluxing agent, which is derived from the thermal decomposition of the borates. Then SiO_2_ generated from the degradation of MVQ was assembled to form a compact integration due to the adhesion of melted B_2_O_3_ after sintering at 1000 °C, resulting in a decrease in the length and volume of the residues. However, the weight loss of the MVQ composites with the addition of different borates is similar, which is because the weight percents of the inorganic fillers such as the borates and halloysites are almost the same.

### 3.5. Surface Morphology of Ceramizable MVQ/HNT Composites and Corresponding Ceramic Residues

**Figure 6:**
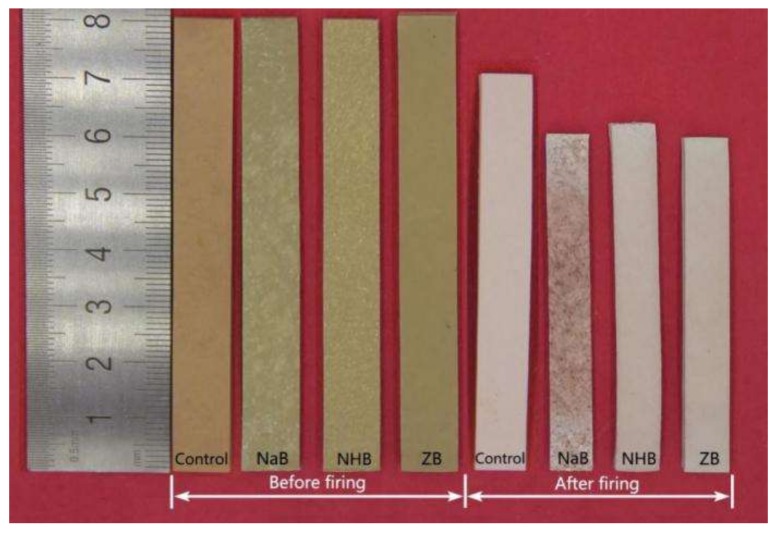
Surface morphology of ceramizable MVQ/HNT composites and corresponding ceramic residues obtained from the pyrolysis products of the composites with the addition of different borates.

The surface morphology of the ceramizable MVQ/HNT composites and corresponding ceramic residues with the addition of different borates was shown in Figure 6. It is exhibited that the control sample and ZB composite is relatively smooth, however, the surfaces of the NaB and NHB composites are rough and dispersed with some white dots. This is because the poor compatibility of sodium tetraborate decahydrate and ammonium pentaborate with MVQ matrix. The corresponding ceramic residues by incorporation of different borates show great linear shrinkage, compared with the control. Moreover, the length of the residue derived from the NHB composite is a little longer than that of another two residues derived from the NaB and ZB composites, respectively. This is because the releasing of ammonia and water stream during the decomposition of ammonium pentaborate leads to an increase in the porosity of ceramic residues and then a decrease in the linear shrinkage. The color of the composites turns from gray (before firing) to pink (after firing), which might be due to some metal oxides such as Fe_2_O_3_ derived from the impurities in HNTs. 

### 3.6. Effect of the Borates on the Flexural and Impact Strength of Ceramic Residues

**Figure 7:**
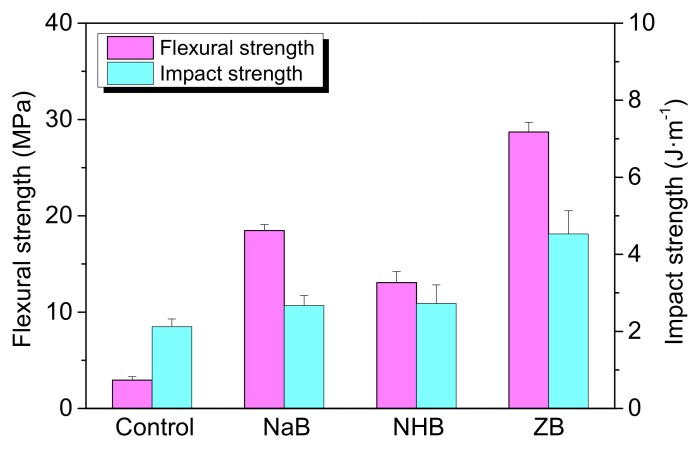
Effect of three borates on the flexural and impact strength of ceramic residues.

Effect of the borates on the flexural and impact strength of ceramic residues was shown in Figure 7. For the control sample, the flexural and impact strength is 2.9 MPa and 2.1 J·m^−1^, respectively. The residues derived from the ZB composites exhibit the maximum flexural and impact strength with the values of 28.7 MPa and 4.5 J·m^−1^, which are 890% and 114% higher than those of the control, respectively. This is because the high thermal stability of zinc borate helps to form dense ceramic structure with improved ceramic strength. However, the residues derived from the NHB composites show the minimum flexural and impact strength with the values of 13 MPa and 2.7 J·m^−1^ respectively, which is due to the releasing of ammonia and crystal water with the decomposition of ammonium pentaborate, destroying the dense structure of the residues.

### 3.7. SEM Analysis

**Figure 8:**
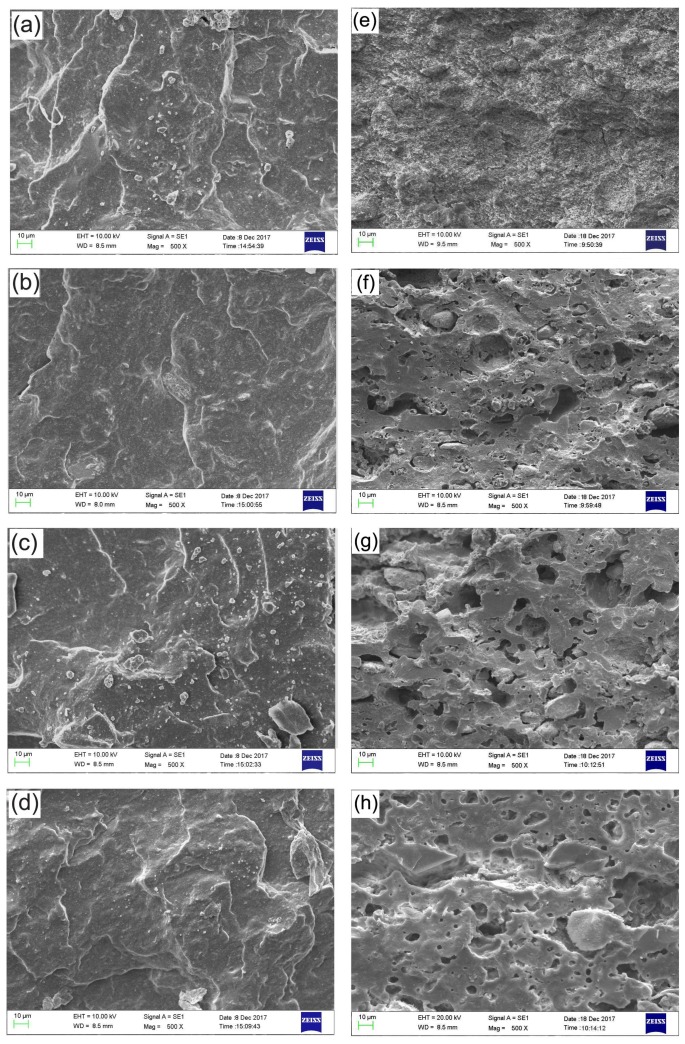
Morphology of tensile fracture surfaces of (**a**) the control and ceramizable, (**b**) NaB, (**c**) NHB, and (**d**) ZB composites and impact fracture surfaces of (**e**) the ceramic residue obtained from the control composite and the ceramic ones derived from the decomposition of the (**f**) NaB, (**g**) NHB, and (**h**) ZB composites, respectively.

The tensile fractures of the control (Figure 8a), ceramizable NaB (Figure 8b), NHB (Figure 8c), and ZB (Figure 8d) composites and the impact fractures of the ceramic residues derived from the decomposition of the composites were shown in Figure 8. Before sintering, the tensile fractures of all the composites are smooth and dense. However, it is found that there are more holes in the impact fractures of the residues containing the borates than that of the control residue (Figure 8e). However, there are fewer cavities in the impact fracture of the ZB residue (Figure 8h) than those of NaB (Figure 8f) and NHB (Figure 8g) residues, indicating that more B_2_O_3_ (liquid phase) generated by the decomposition of zinc borate makes the ceramic residues denser. The releasing of more crystalliferous water or ammonia accompanied by the decomposition of sodium tetraborate decahydrate and ammonium pentaborate might result in many holes in the fracture surface. 

### 3.8. XRD Analysis

**Figure 9:**
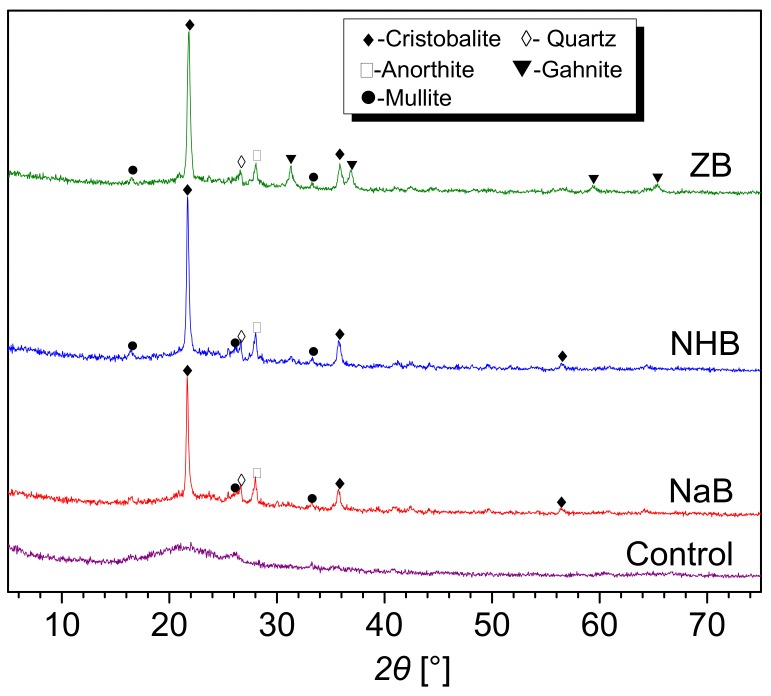
XRD spectra of the ceramic residues obtained from the MVQ/HNT composites with the addition of different borates.

XRD spectra of the ceramic residues obtained from the decomposition of the NaB, NHB, and ZB composites after sintering at 1000 °C was shown in Figure 9. The residues without any borates were chosen as control. For the control residue, there is no diffraction peaks in the XRD spectrum, indicating no crystalline phases existed in the residues. For the residues with the addition of the borates, the main crystalline phases [25,26] include quartz (SiO_2_) (JCPDS no. 46-1045) and cristobalite (JCPDS no. 76-1390), anorthite (CaAl_2_Si_2_O_8_) (JCPDS no. 41-1486) and mullite (Al_6_Si_2_O_13_) (JCPDS no. 15-0776). It is known that amorphous silica (SiO_2_) was obtained from pyrolysis of MVQ, and alumina (Al_2_O_3_) was obtained from the decomposition of halloysites and calcium oxide (CaO) was obtained from the degradation of calcium carbonate (CaCO_3_) in the sintering process. Because the eutectic reactions were triggered within SiO_2_, Al_2_O_3_, and CaO, some new crystals such as cristobalite, quartz, anorthite, and mullite emerged after cooling of the residues. However, for the residue derived from the ZB composite, the diffraction peaks (*2*θ = 31.227°, 36.772°) appear in the XRD spectra, indicating the generation of gahnite (ZnAl_2_O_4_) (JCPDS no. 05-0669), which is due to the eutectic reaction between ZnO and Al_2_O_3_.

### 3.9. Proposed Ceramizable Mechanism

The decomposition of three berates and proposed eutectic reactions between different metal oxides are shown in Equations (3)–(14). According to the TGA results in Figure 2, sodium tetraborate decahydrate (Na_2_B_4_O_7_·10H_2_O) is white crystal, which will be decomposed to B_2_O_3_ at elevated temperature. Ammonium pentaborate (NH_4_B_5_O_8_·4H_2_O), which is decomposed to B_2_O_3_ at the temperature up to 470 °C. Zinc borate (2ZnO·3B_2_O_3_·3.5H_2_O) can act as an inorganic boron-containing flame retardant, which is decomposed to B_2_O_3_ from 370 to 450 °C [20]. The thermal decomposition of halloysites involves dehydroxylation at 500~900 °C, the silica and alumina are originally formed in the tetrahedral and octahedral sheets, respectively. Nano sized (5–40 nm) γ-Al_2_O_3_ is generated at 900~1000 °C [27]. There are eutectic reactions between some metal oxides such as Al_2_O_3_, SiO_2_, CaO, and ZnO to generate anorthite (CaAl_2_Si_2_O_8_), mullite (Al_6_Si_2_O_13_), gahnite (ZnAl_2_O_4_), etc. All the crystals will strengthen the ceramic residues and increase their flexural and impact strength [28].

Decomposition of sodium tetraborate decahydrate:

70~100 °C        Na_2_B_4_O_7_·10H_2_O → Na_2_B_4_O_7_+10H_2_O↑ (3)

100~500 °C    Na_2_B_4_O_7_ → 2NaBO_2_+B_2_O_3_ (4)

Decomposition of ammonium pentaborate:

100~200 °C      NH_4_B_5_O_8_·4H_2_O → NH_4_B_5_O_8_·H_2_O+3H_2_O↑ (5)

200~350 °C       NH_4_B_5_O_8_·H_2_O → HB_5_O_8_+H_2_O↑+NH_3_↑ (6)

350~470 °C            2HB_5_O_8_ → 5B_2_O_3_+H_2_O↑ (7)

Decomposition of zinc borate:

370~500 °C  2ZnO·3B_2_O_3_·3.5H_2_O → 2ZnO+3B_2_O_3_+3.5H_2_O↑ (8)

Transformation of silica:

1000 °C  SiO_2_ (amorphous) → SiO_2_ (crystal) (9)

Decomposition of halloysites:

500~900°C Al_2_(OH)_4_Si_2_O_5_·2H_2_O (Halloysites) → Al_2_O_3_·2SiO_2_+4H_2_O↑ (10)

900~1000°C  Al_2_O_3_·2SiO_2_ → Al_2_O_3_+2SiO_2_ (11)

Proposed eutectic reactions:

2SiO_2_ + 3Al_2_O_3_ → Al_6_Si_2_O_13_ (mullite) (12)

CaO + 2SiO_2_ + Al_2_O_3_ → CaAl_2_Si_2_O_8_ (anorthite) (13)

ZnO+ Al_2_O_3_ → ZnAl_2_O_4_ (gahnite) (14)

## 4. Conclusions

The mechanical and electrical properties of ceramizable silicone rubber (MVQ)-based composites with the addition of zinc borate are better than those of sodium tetraborate decahydrate and ammonium pentaborate. The tensile strength of MVQ/HNT composites with zinc borate is 69% and 42% bigger than that with sodium tetraborate decahydrate and ammonium pentaborate, respectively. The volume and surface resistivity of MVQ/HNT composites with zinc borate are higher than those with sodium tetraborate decahydrate and ammonium pentaborate, showing the values of 1.7 × 10^14^ Ω·m and 7.9 × 10^14^ Ω, respectively. The flexural and impact strength of the ceramic residues derived from decomposition of the composites by incorporation of zinc borate are 28.7 MPa and 4.5 J·m^−1^ respectively due to the dense structure in the residues. Furthermore, the eutectic reactions between zinc borate and halloysite will generate some crystals such as mullite and gahnite in the residues after sintering at 1000 °C, which further increases the flexural and impact strength of the residues.

## Figures and Tables

**Table 1:** The formulations of ceramizable MVQ/HNT composites.

Ingredients (phr)	Symbols			
	Control	NaB	NHB	ZB
Silicone rubber	100	100	100	100
Fumed silica	40	40	40	40
Halloysite	30	30	30	30
Calcium carbonate	5	5	5	5
Sodium tetraborate decahydrate	-	7.5	-	-
Ammonium pentaborate	-	-	7.5	-
Zinc borate	-	-	-	7.5
DBPMH	2	2	2	2

**Table 2:** Thermogravimetric analysis of NaB, NHB, and ZB fillers.

Samples	Initial Degradation Temperature, T_5%_ (°C)	Maximum Degradation Temperature, T_d%_ (°C)	Mass of Residue at 900 °C (%)
NaB	66.9	84.0	54.2
NHB	144.0	184.0	64.6
ZB	371.5	417.0	84.3

Note: NaB: sodium tetraborate decahydrate; NHB: ammonium pentaborate; ZB: zinc borate.
